# Can Asymmetrical Mechanical Loading Be Accurately Inferred From the Analysis of Skeletal Material?

**DOI:** 10.1002/ajpa.70176

**Published:** 2025-12-06

**Authors:** Antonio Profico, Nathan Jeffery, Fotios Alexandros Karakostis

**Affiliations:** ^1^ Department of Biology University of Pisa Pisa Italy; ^2^ Department of Musculoskeletal & Ageing Science, Institute of Life, Course and Medical Sciences University of Liverpool UK; ^3^ Palaeoanthropology, Department of Geosciences University of Tübingen Tübingen Germany; ^4^ Senckenberg Centre for Human Evolution and Palaeoenvironment Senckenberg Research Institute Tübingen Germany; ^5^ Integrative Prehistory and Archaeological Science University of Basel Basel Switzerland

**Keywords:** bilateral asymmetry, handedness, limb preference, physical activity, virtual anthropology

## Abstract

**Objectives:**

Reconstructing habitual limb preference in the past is crucial for understanding the evolution of hominin behavior. However, our ability to reliably identify asymmetrical behaviors from bone remains is limited due to a lack of experimental evidence directly correlating a history of loading asymmetry with skeletal asymmetry. We address this gap by analyzing an existing laboratory sample of rats subjected to asymmetric loading, relying on four methodological approaches that address both external and internal bone morphology.

**Materials and Methods:**

Data were derived from nine genetically consistent Wistar rat tibiofibulae, exposed to controlled asymmetrical loading. Asymmetry was evaluated using 3D geometric morphometrics for analyzing bone shape, cross‐sectional geometry for assessing biomechanical strength, cortical thickness mapping of compact bone distribution, and V.E.R.A. (1.0 and 2.0) for quantifying 3D muscle attachment sites.

**Results:**

The findings revealed a clear association between asymmetrical loading and bone asymmetry, particularly in the distal periosteum and medio‐anterior midshaft, which exhibited notable 3D shape changes and increased cortical thickness. Additionally, polar second moment of area values were higher in stimulated limbs, indicating improved biomechanical function. The 3D entheseal areas of the stimulated limbs were also proportionally larger, with no apparent association with allometric factors.

**Discussion:**

This study provides experimental proof‐of‐concept that asymmetric biomechanical loading influences skeletal bilateral asymmetry, suggesting that reconstructing limb preference is feasible using these methods. Future applications could enhance our understanding of the evolution of hominin handedness and its role in ancient lifeways.

## 1 Introduction

Bone tissue is a dynamic and adaptable tissue that can, within certain physiological constraints, respond in an orderly fashion to mechanical loads placed on it during an individual's life. Thus, in theory, the loading history of an individual can be reconstructed from the study of osteological remains by biological anthropologists, using a backward engineering approach. The underlying principle is known as “Wolff's law,” which states that bone tissue adapts its structure in response to habitual mechanical loads (Barak et al. 2011; Pearson and Lieberman 2004; Wolff 1986). The modeling can take place by parsimoniously varying bone density, size and shape to effectively resist the mechanical loading with the minimum amount of bone material. More recently, these ideas have been expanded upon in the “bone functional adaptation” concepts, which emphasize the complexity and nuance of bone adaptation processes (Ruff et al. 2006). These modern frameworks build on Wolff's law by incorporating factors such as the timing, intensity, and type of mechanical stimuli, reflecting a deeper understanding of bone's response to diverse and changing biomechanical environments.

Applications in biological anthropology typically include the comparison of cortical bone distribution at homologous levels (e.g., a cross section at the mid‐shaft), the analysis of skeletal shape (e.g., geometric morphometrics) and the investigation of the muscle insertion sites (e.g., musculoskeletal markers, including entheseal changes) (Karakostis 2022, 2025a; Profico, Bondioli, et al. 2021; Ruff et al. 2006; Schrader 2019; Shaw and Stock 2009). One of the applications of bone modeling in response to mechanical loading is the analysis of the pattern of asymmetry of antimeric skeletal elements (e.g., Steele and Mays 1995). Morphological asymmetry of long bones has been hypothesized to be linked to side preference (e.g., Barut et al. 2011; Plato et al. 1980; Roy et al. 1994; Steele and Mays 1995). This hypothesis is supported by the analysis of antimeric bones in athletes (playing arm vs. not playing arm) compared with individuals classified into different categories of loading history (Shaw and Stock 2009).

In biological anthropology different methodological approaches are used to infer the asymmetry of loading history from the analysis of long bone morphology (e.g., Buikstra and Beck 2017; Buikstra 2024; Karakostis and Harvati 2021; Lieberman et al. 2004; Profico, Zeppilli, et al. 2021; Ruff et al. 2006). These techniques include geometric morphometrics, cross‐sectional geometry, cortical bone distribution and areas of muscle insertion sites. Geometric Morphometrics (GM) involves the acquisition of Cartesian coordinates at homologous anatomical landmarks to analyze shape variations (e.g., Adams et al. 2013; Mitteroecker and Gunz 2009). Landmark data are converted into shape variables after removing information about position, rotation and size. Subsequently, shape variables can be used to analyze both symmetric and asymmetric components. Another, earlier method, is inspired by the classical beam theory, proposed by (Timoshenko 1921). This method is commonly used by anthropologists to predict the mechanical properties of long bones by analyzing their cross‐sectional geometry (CSG) along the diaphysis (Lovejoy et al. 1976; Ruff 1981). Cross‐sections are extracted along the biomechanical length of the bone and used to calculate area moments of inertia, section moduli, and shape indices. The application of geometric morphometrics on cross‐sections has led to the development of a new method to investigate the pattern of distribution of cortical bone thanks to the calculation of distances between paired equidistant geometric coordinates on the periosteum and endosteum (Profico, Bondioli, et al. 2021). More recently, the “Validated Entheses‐based Reconstruction of Activity” (V.E.R.A.) 1.0 and 2.0 methods have been developed and recommended for reconstructing past physical behavior through univariate and/or multivariate analysis of precisely quantified 3D muscle attachment areas (Karakostis and Lorenzo 2016; Karakostis 2025a; see dedicated literature reviews by Karakostis and Harvati 2021; Karakostis 2022).

However, despite recent experimental animal studies supporting the reliability of certain approaches for reconstructing habitual loading and physical activity (e.g., Castro et al. 2022; Karakostis, Jeffery, and Harvati 2019; Karakostis, Wallace, et al. 2019; Karakostis and Wallace 2023; Karakostis 2025a; Turcotte et al. 2022), no experimental study has yet confirmed the capacity of these methods to infer habitual limb preference from skeletal remains. This significant research gap likely arises from the inherent difficulties in designing experiments that accurately simulate habitual limb preference in nonhuman laboratory animals. As a result, it remains uncertain whether it is even possible, in principle, to reliably identify habitual limb preference in osteological contexts.

In this experimental study, we aim to address these questions by applying 3D geometric morphometrics, cross‐sectional geometry, morphometric mapping of relative cortical thickness, and the V.E.R.A. methods on an osteological sample of rats. These laboratory rats, drawn from the same strain (Wistar), biological sex and of the same age, were subjected to controlled conditions of asymmetrical mechanical loading. This rare experimental setup provides a unique opportunity to investigate the effects of asymmetrical loading under controlled conditions, to our knowledge, for the first time.

## 2 Materials and Methods

The sample consists of bilateral microtomographic scans of the tibiofibular complex in nine eight‐week‐old male Wistar rats from a previous in vivo experiment described in Vickerton et al. (2014). In six out of nine rats, the left common peroneal nerve was repeatedly activated by electrical stimulation, inducing a near maximum force muscle contraction every 30 s for 28 days. Scan resolution was 40 μm isotropic, and 3D volumes were reconstructed using a standardized isosurface threshold. In the previous study, Vickerton et al. (2014) performed a Finite Element Analysis on the tibiofibula, simulating strain distributions between stimulated and unstimulated hindlimbs and conducting bone histological and morphometric analyses. Here, we further investigated this collection of scans by analyzing the shape and the cortical distribution of the tibiofibular diaphysis, the muscle attachment areas, and biomechanical parameters along the diaphysis.

**FIGURE 1:**
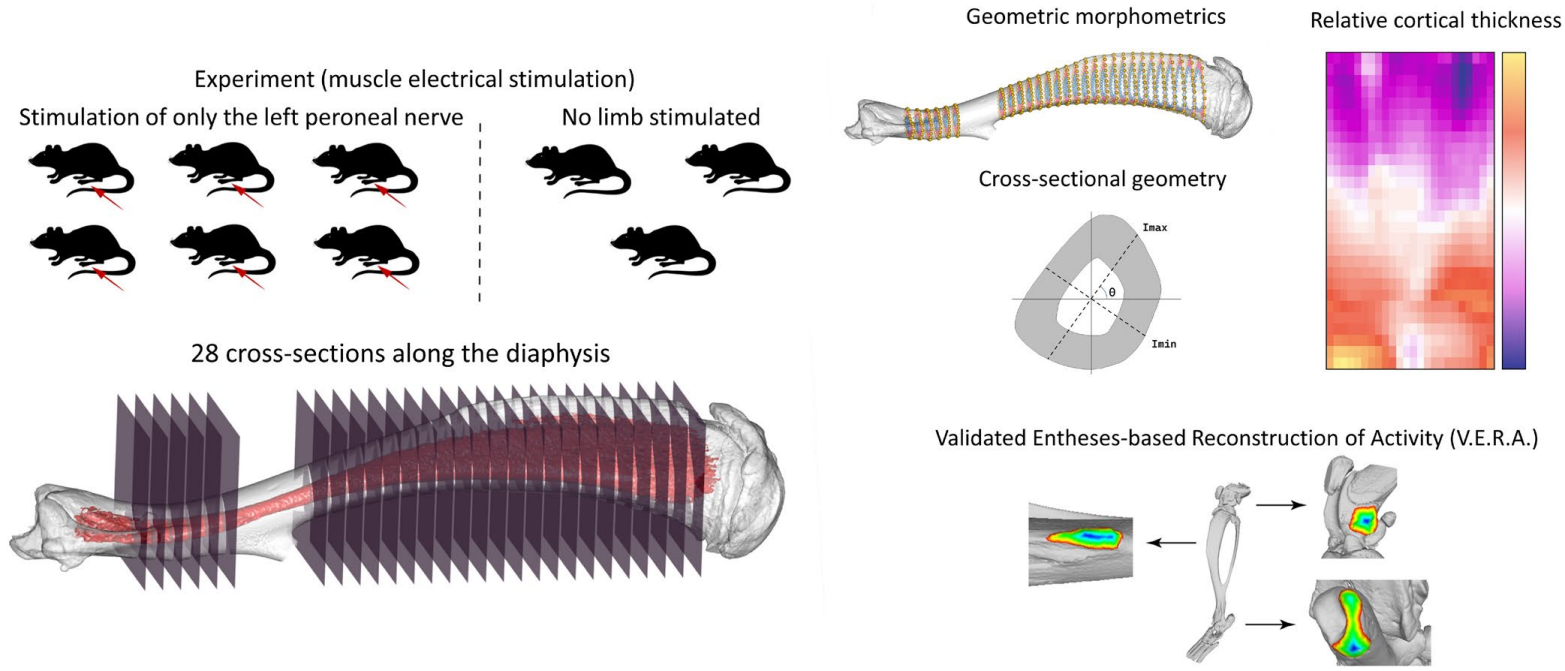
Sample of study and extraction of the cross‐sections from the diaphysis. Cross‐sections and the 3D models are used to apply geometric morphometrics, cross‐sectional geometry, relative cortical thickness mapping and the V.E.R.A. methods. The cross‐sections in correspondence with the fusion between tibia and fibula have been excluded from the analysis.

The eighteen 3D models of tibiofibular have been aligned following standard protocols (Ruff 2002). On each left and right tibiofibula, we extracted 28 cross‐sections along the diaphysis in two length intervals: 6 cross‐sections from 12.5% to 25.0% and 22 cross‐sections from 37.5% to 90%. The cross‐sections from 25.0% to 37.5% had been excluded because they correspond to the region where tibia and fibula elements are fused (Figure 1).

### 2.1 3D Geometric Morphometrics

We applied the R functions available in the *morphomap* R package (Profico, Bondioli, et al. 2021) to extract from each cross‐section 24 pairs (periosteum and endosteum) of equiangular semilandmarks centred at the geometric barycentre of the cross‐section. The GM dataset for each tibia consists of 1344 equiangular semilandmarks (24 semilandmark × 2 × 28 sections).

The configurations of semilandmark coordinates were translated, rotated and scaled using generalized Procrustes analysis (GPA). We explore the main axes of variation on shape variables by applying Principal Component Analysis (PCA) and we calculate the percentage of variance related to mechanical loading (stimulated hindlimbs).

### 2.2 Targeted Geometric Morphometrics

**FIGURE 2:**
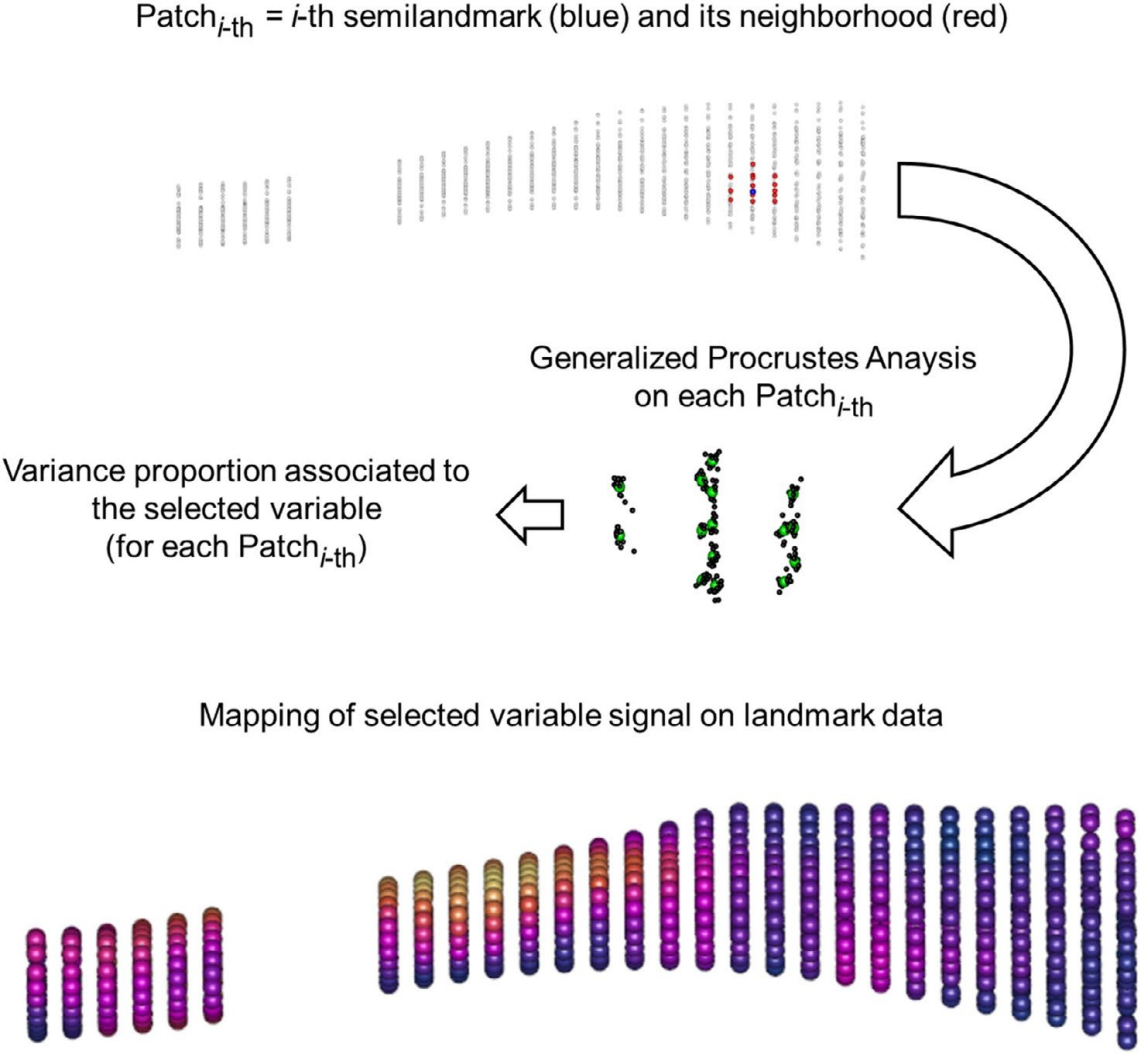
Workflow summarizing the production of 3D color maps associated with the variable “stimulation”. The cross‐sections in correspondence with the fusion between tibia and fibula have been excluded from the analysis.

We extended the geometric morphometric analysis by designing a protocol to subset from the entire dataset of semilandmarks only the regions that were statistically correlated to the categorical variable “stimulation.” In contrast to a traditional GM approach, the entire set of semilandmarks is analyzed locally by selecting a subset of points. In detail, on each of the 1344 semilandmarks (landmark*‐th*), a neighborhood of 15 semilandmarks (N‐core) is defined. The same N‐core is selected on each tibia, and a GPA step is performed. On the shape variables of N‐core, the percentage of variance associated with the binary categorical variable stimulation/no stimulation is calculated. The iterative process is repeated 1344 times, and the average value of the variance proportion associated with the variable *stimulation* is built and converted to a 3D color map (Figure 2) (Del Bove et al. 2023). This approach allows us to select a subset of local portions from the entire shape that were associated with a given variable (stimulation in this study). Subsequently, we converted the selected points into shape variables by performing a GPA in the shape space.

### 2.3 Cross‐Sectional Geometry

We relied on the package *morphomap* to calculate from the 28 cross‐sections of each tibia cross‐sectional geometry (CSG) parameters (total areas, endosteal area, cortical area, total perimeter, endosteal perimeter, Imin, Imax, J). The CSG dataset for each tibia is composed of 224 variables (28 cross‐sections × 8 CSG parameters). CSG values have been standardized using the total biomechanical length as a proxy for an individual's body size (Ruff 2000). Standardized CSG data has been analyzed by using principal components and the mechanical pattern of variations calculated at the extreme of the first two PC scores.

### 2.4 Relative Cortical Thickness Distribution

We built matrices of relative cortical thickness by calculating the linear distances between pairs of 1000 equiangular semilandmarks (cortical and the endosteal sets) over the distance between the periosteal semilandmarks and the center of gravity of the cross‐section. The matrix represents the values of cortical thickness adjusted by the radius of the cross‐section. The matrices of relative cortical thickness have been analyzed by PCA and the morphometric map variations calculated at the extremes of the first two PC scores by using the functions *morphomapPCA* and *morphomapVariations*.

### 2.5 V.E.R.A. Methods (1.0 and 2.0)

The 3D surface areas (in mm^2^) of three muscle attachment structures on the rat lower limb bones were derived from previous research (Karakostis, Jeffery, and Harvati 2019), where they had been calculated using the standard V.E.R.A. 1.0 method, first introduced in 2016 (Karakostis 2022; Karakostis and Harvati 2021; Karakostis and Lorenzo 2016). These include the calcaneal tuberosity (corresponding to muscles not stimulated in the experiment) and two entheses‐bearing skeletal structures corresponding to muscles stimulated, including the origin attachment regions of *tibialis anterior* (TA) and *extensor digitorum longus* (EDL) on the tibia. Full methodological details are provided in the original study. In brief, the process involved creating iso‐surface scans through segmentation of micro‐CT data, with V.E.R.A. 1.0 then used to delineate the borders of each structure on the bone surface based on the criteria of relative elevation and irregularity, and relying on the sequential use of various curvature filtering algorithms in the software Meshlab (Callieri et al. 2012). The resulting measurements are subjected to a PCA that relies on the correlation matrix. It should be clarified that, in the present study, 3D entheseal measurements were not adjusted for individual size using the geometric mean approach (as applied in the previous study; see also Karakostis 2022), in order to capture a more comprehensive measure of bilateral asymmetry based on actual entheseal 3D sizes (rather than size‐normalized values based on each individual's geometric mean). Nevertheless, the resulting distinction between stimulated and non‐stimulated specimens still closely mirrored the pattern observed in the earlier study.

**FIGURE 3:**
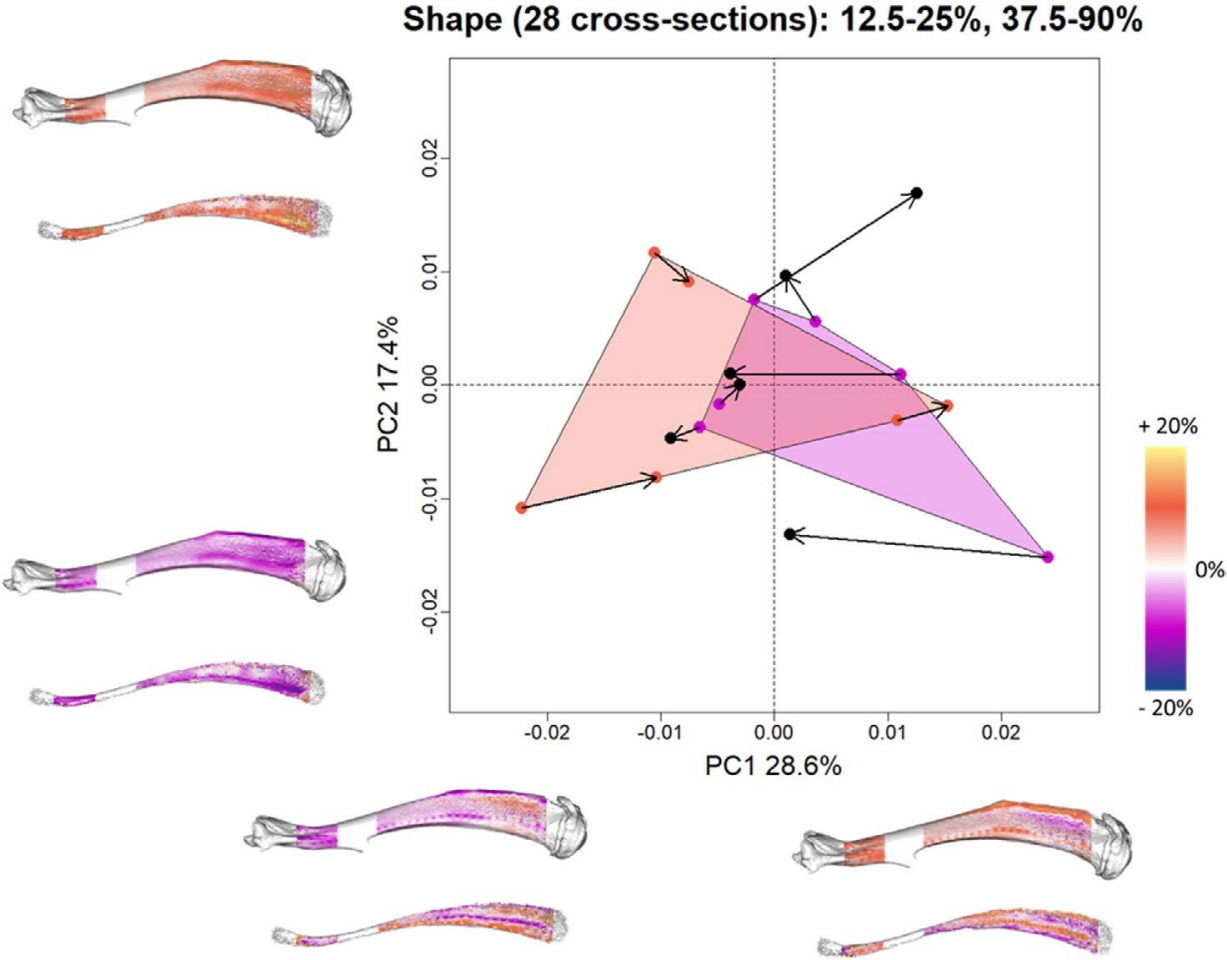
Principal component plot of the diaphyseal shape. Stimulated rat hindlimbs are reported in magenta, control groups in dark orange. The arrows represent links from left to the right side. Shape variations are reported at the extreme values of PC1 and PC2. Warm and cold color palettes represent respectively the region characterized by local expansion and reduction of surface area.

In addition to the V.E.R.A. 1.0 approach, which examines the 3D surface area of entire bone structures (such as tuberosities and ridges), we also carried out a supplementary, targeted analysis of entheseal asymmetry using the newly developed V.E.R.A. 2.0 method (Karakostis 2025a; Karakostis 2025b). While V.E.R.A. 1.0 quantifies complete enthesis‐bearing regions on the bone surface, including entire tubercles or ridges, the 2nd version enables the semi‐automatic identification and measurement of entheseal changes (specifically, surface irregularities or deformations) that are arguably considered more directly associated with cumulative muscle‐induced biomechanical loading (Karakostis 2025a; see also discussions in Karakostis and Wallace 2023). Previous experimental studies have demonstrated that V.E.R.A. 2.0 can reconstruct habitual activity patterns with comparable accuracy to V.E.R.A. 1.0, both under controlled laboratory conditions and in human skeletons with extensively documented occupational histories. Notably, it achieves this with reduced analytical effort and often requires fewer entheses to be examined (Karakostis 2025a). For this supplementary analysis, we focused on entheseal changes within the ridge associated with the TA muscle, which was stimulated during the experiment and also played a key role in the activity‐related differences identified by V.E.R.A. 1.0 (Karakostis, Jeffery, and Harvati 2019). The broader selection region included the entire distinctive ridge structure; for example, see area indicated in (Karakostis, Wallace, et al. 2019) (Figure 3). The percentage difference between left and right specimens was then calculated and visualized using a standard jitter plot.

### 2.6 Statistical Analysis

All statistical analyses were conducted in R (R Core Team 2021). For multivariate datasets, PC scores were selected until reaching 90% of the cumulative variance. Statistical significance was defined at *α* = 0.05, and all reported *p*‐values from ANOVA and multivariate regressions were obtained via permutation tests. The assumption of multivariate normality was evaluated using Royston's multivariate normality test (Royston 1983).

## 3 Results

### 3.1 Geometric Morphometrics

The PCA plot of the first two PC scores is associated with 45.99% of the total variance (Figure 3). The control group (no stimulated hindlimbs) is placed mainly at negative values of PC1 and around neutral values of PC2. On the contrary, the stimulated hindlimbs mainly segregate at positive values of PC1 and around neutral values of PC2. The two groups are largely overlapped. At positive values of PC1, the distal portion of the diaphysis is characterized by an increase in periosteal and endosteal surface. On the mid‐proximal part of the diaphysis, at positive values of PC1, the anterior and lateral margins are characterized by a contraction of the surface area. On the contrary, the region between the anterior and lateral borders is marked by a reduction in surface area. The opposite pattern is associated with negative values of PC1. PC2 is linked to a more general pattern, with increases in surface area at positive values and decreases at negative values (Figure 3). The multivariate regression of the entire set of PC scores highlights the absence of a statistically significant relation with the variable “Stimulation” (*R* ^2^ = 0.11, *p*‐value = 0.057). However, the ANOVA performed on single PC scores revealed that PC4 (9.95%) is significantly (*R* ^2^ = 0.46, *p*‐value = 0.002) associated with the variable “Stimulation” (Figure S1).

**FIGURE 4:**
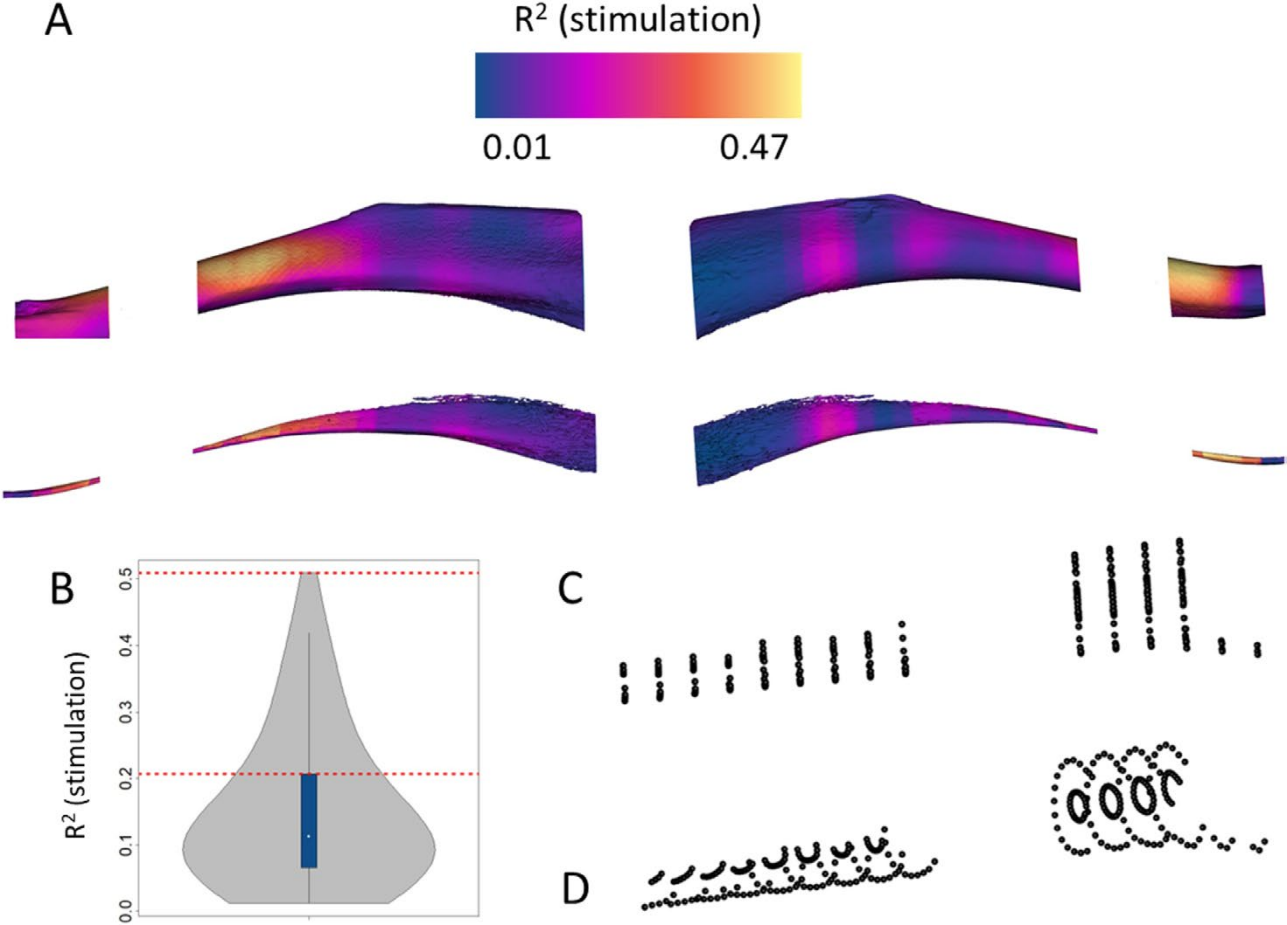
Targeted PCA: Definition of semilandmark dataset. The average proportion of variance per semilandmark associated with the variable “stimulation” (a) is used to calculate the third quartile (b) of the distribution and to define a subset of the entire set of semilandmarks (c and d).

Targeted PCA is a novel approach designed to measure and quantify the association of local regions with a numerical or categorical variable (Del Bove et al. 2023; Sansalone et al. 2023). Here, we report the results of the mapping of the variable “Stimulation” on the set of semilandmarks after Procrustes registration. The statistical relationship between shape and variable “Stimulation” is ranged between 0.01 and 0.47 over the entire set of semilandmarks (Figure 4). We define as a threshold the third quartile that is equal to 0.22. The semilandmarks showing on average a value of *R* ^2^ (coefficient of determination from linear regression model) equal to or higher than 0.22 are reported in Figure 4C,D and they constitute the targeted set.

**FIGURE 5:**
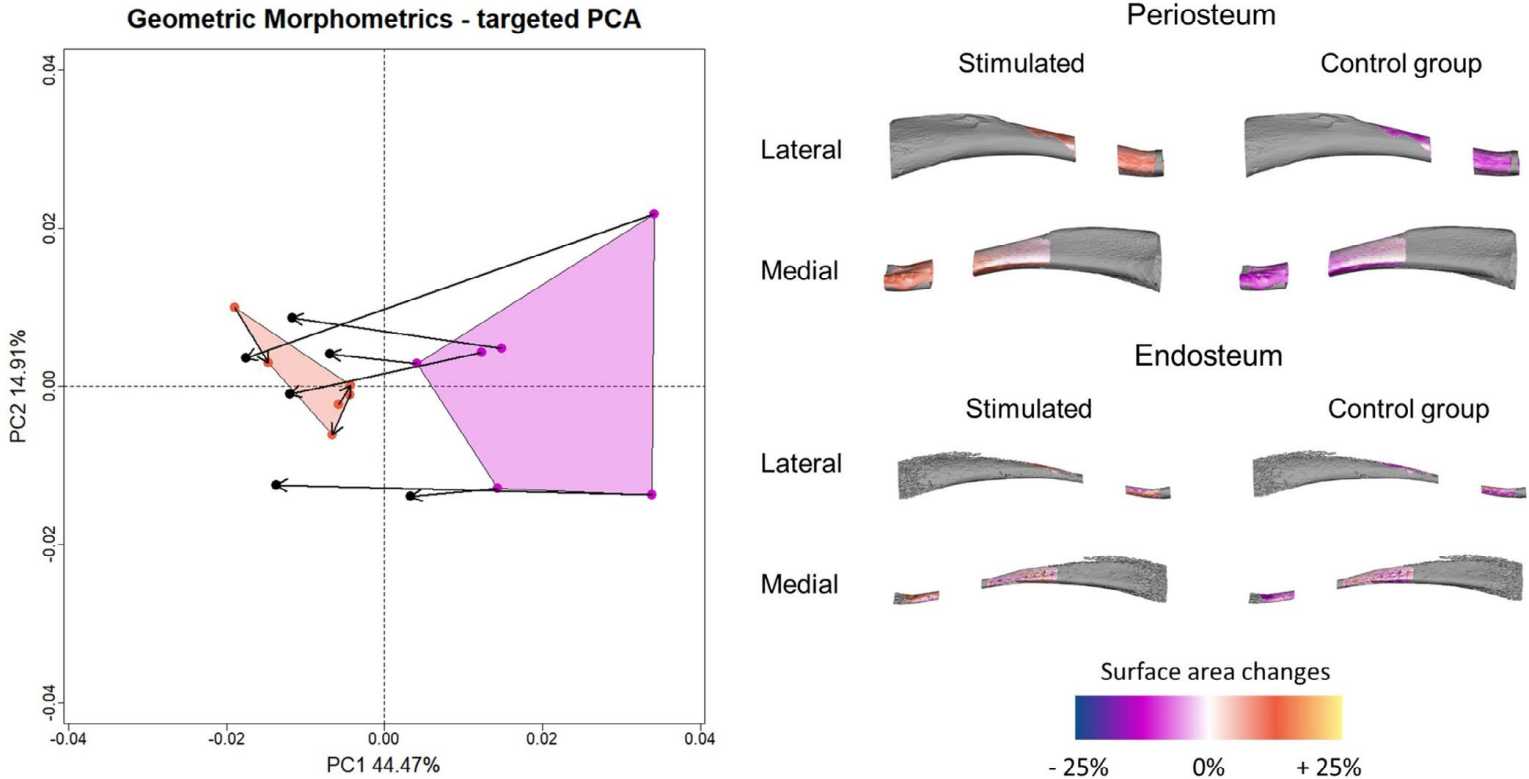
Targeted principal component analysis. Stimulated rat hindlimbs are reported in magenta, control groups in dark orange. The arrows represent links from left to the right side. Shape variations are reported at the extreme values of PC1 and PC2. Warm and cold color palettes represent respectively the region characterized by local expansion and reduction of surface area.

The first two PC scores of the PCA performed on the targeted set explain respectively 44.47% and 14.91% of the total variance. The two groups are clearly distinguished along the PC1. In the stimulated group, the right hindlimbs (not stimulated) fall outside the group of the left hindlimbs (stimulated) close to the control group (left and right hindlimbs not stimulated). Stimulated hindlimbs are characterized by a contraction of the area of the periosteal surface of the distal portion of the diaphysis. The endosteum is expanded anteriorly and posteriorly, while reduced medio‐laterally (Figure 5). On the segment comprised between the proximal and middle regions of the diaphysis the anterior and posterior parts of the periosteum are expanded. The lateral facet of this portion of the diaphysis is characterized by an expansion of the distal portion and contraction of the most proximal portion. The endosteum is mainly characterized by a reduction of the surface area.

The multivariate regression of the set of PC scores highlights a statistically significant relation with the variable “Stimulation” (*R* ^2^ = 0.36, *p*‐value = 0.001).

### 3.2 Cross‐Sectional Geometry

**FIGURE 6:**
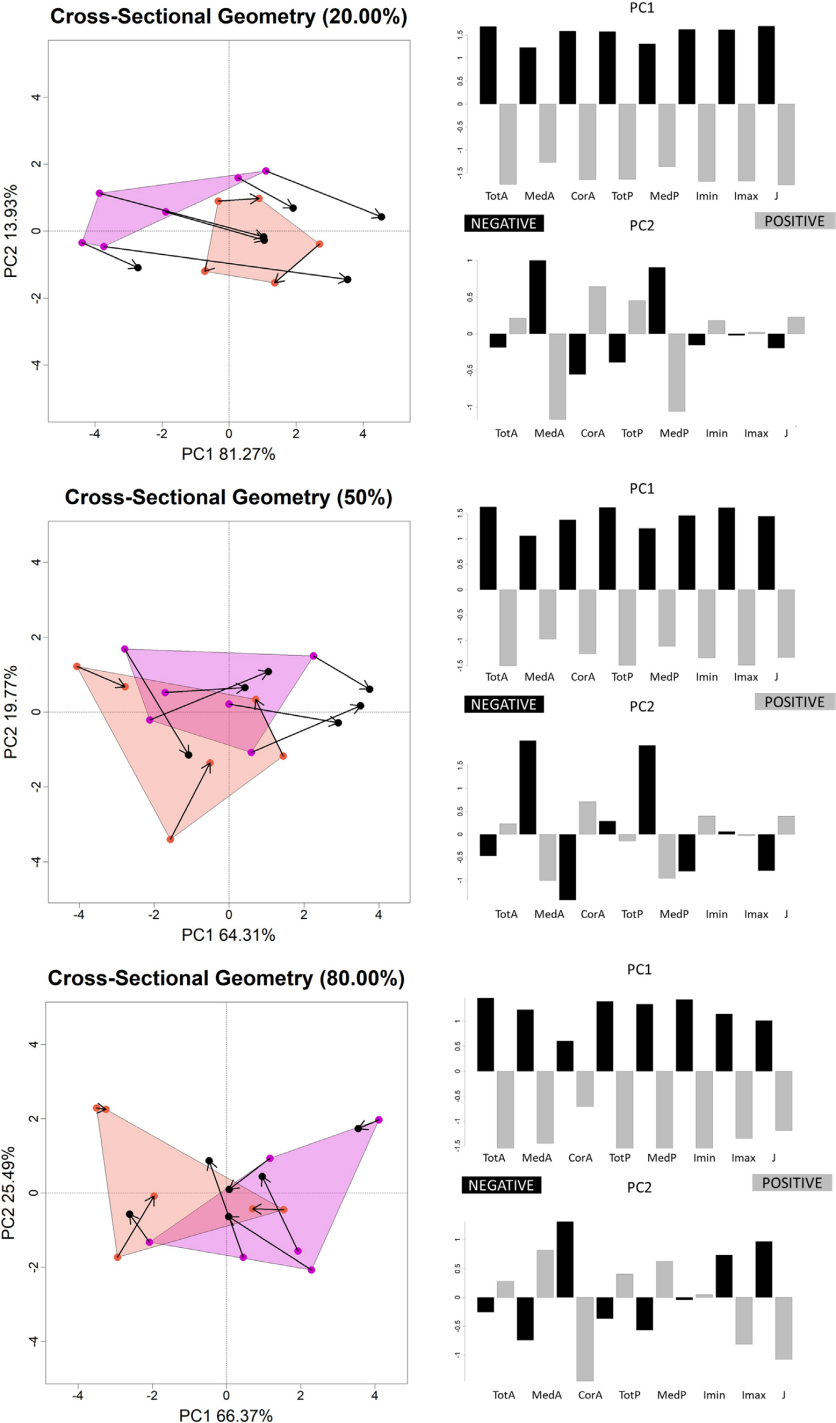
Principal component analysis on cross‐sectional geometry parameters. Stimulated rat hindlimbs are reported in magenta, control groups in dark orange. The arrows represent links from left to the right side. Principal component loadings are calculated at the extreme values of PC1 and PC2, based on cross‐sections at the distal (20%), middle (50%), and proximal (80%) portions of the diaphysis. TotA = total area, MedA = medullary area, CorA = cortical area, TotP = total perimeter, MedP = medullary perimeter, Imin = minimum moment of inertia, Imax = maximum moment of inertia, J = polar moment of inertia.

The values of cross‐sectional geometry (total area, endosteal area, cortical area, total perimeter, endosteal perimeter, Imin, Imax, J) of three cross‐sections at 20% (distal region), 50% (mid‐shaft) and 80% (proximal regions) have been analyzed by applying PCA (Figure 6).

The distal subset (Table S1) shows a distribution along the PC1 according to the stimulation variables. The non‐stimulated limbs, compared to the stimulated hindlimbs, are shifted toward positive values of PC1 where the control group is located. The stimulated hindlimbs are placed along negative values of PC1. Loadings of PC1 reflect the increment of all the CSG parameters at negative values of PC1. The multivariate regression of PC scores shows a statistically significant relation with the variable “Stimulation” (*R* ^2^ = 0.34, *p*‐value = 0.007). PC2 records variations of biomechanical parameters associated with an enlargement of medullar area at negative values. At positive values of PC2 there is an increase in cortical area, total area and J value.

At the midshaft (Table S2), along both PC1 and PC2 there is no separation between the control group and the hindlimbs of the stimulated rats. PC1 is linked to a general increase in biomechanical parameters at negative values. PC2 is associated with an enlargement of medullary area at negative values and with an increase in cortical area and J at positive values. The multivariate regression of the PC scores shows no significant relation with the variable “Stimulation” (*R* ^2^ = 0.15, *p*‐value = 0.090).

At the proximal region (Table S3) of the shaft, there is a slight separation along PC1 between stimulated rats and the control group. PC1 records a general increase of biomechanical parameters at negative values. PC2 is associated with a reduction of cortical area, Imax, and J at positive values. The multivariate regression of the PC scores shows not a significant relation with the variable “Stimulation” (*R* ^2^ = 0.05, *p*‐value = 0.466).

### 3.3 Relative Cortical Bone Distribution

**FIGURE 7:**
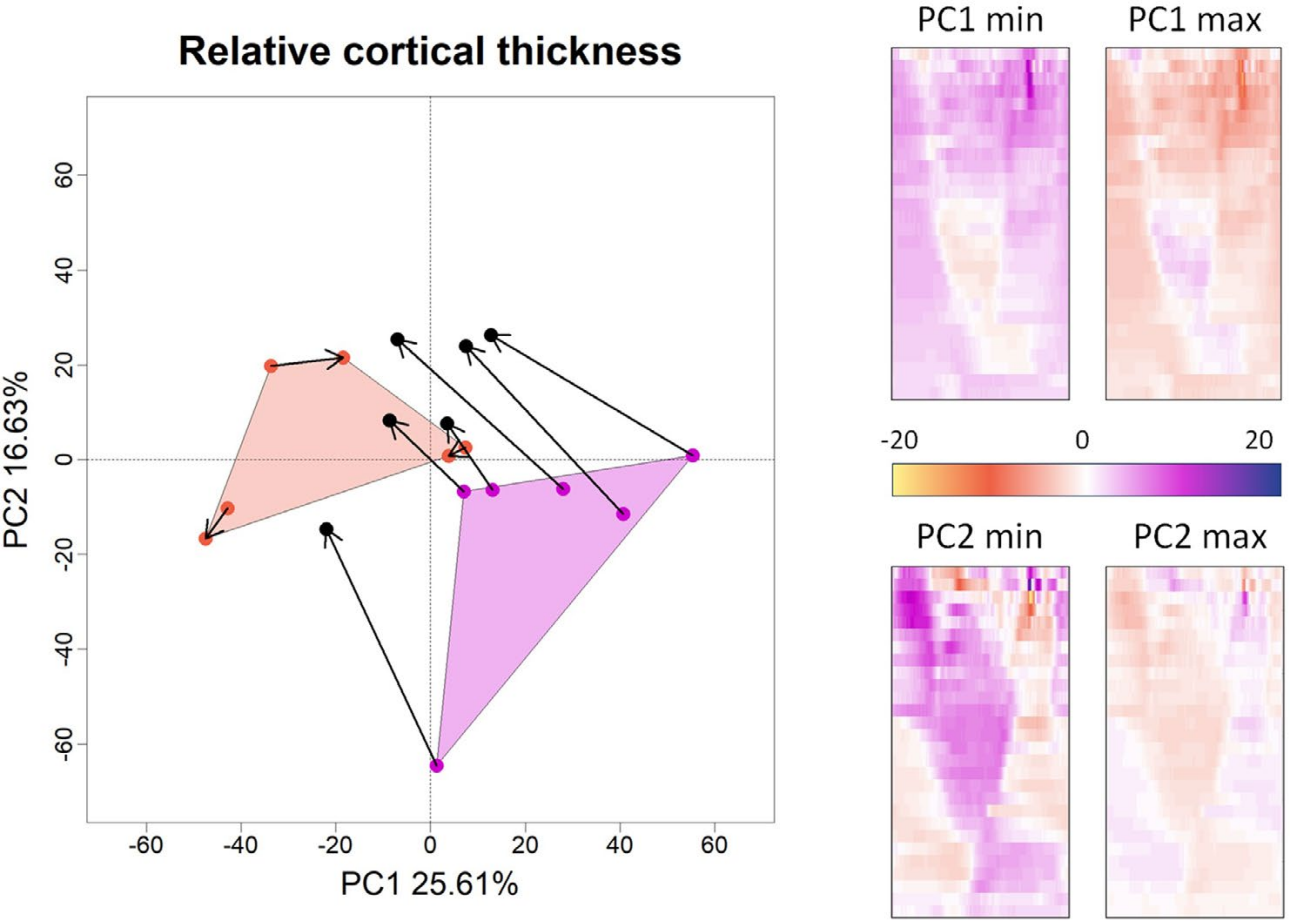
Principal component analysis on relative cortical maps of the entire diaphysis. Stimulated rat hindlimbs are reported in magenta, control groups in dark orange. The arrows represent links from left to the right side. On the right are reported the relative cortical map variations calculated at the extreme values of the first two PC scores. Warm and cold color palettes represent respectively the region characterized by a relative increment and decrement of cortical thickness.

The distribution of the cortical bone has been investigated by measuring the relative thickness across the diaphysis between 12.5% and 90% of the biomechanical length after excluding the portion where the fibula and tibia are fused (between 25% and 37.5%). The first two PC scores of the PCA performed on 2D maps of relative cortical thickness are associated, respectively, with 25.61% and 16.63% of the total variance (Figure 7). The multivariate regression of the PC scores (up to 90% of the variance) highlights a statistically significant relation with the variable “Stimulation” (*R* ^2^ = 0.19, *p*‐value = 0.001).

Rats of the control group (negative values of PC1) are well separated from the stimulated rats (positive values of PC1). In addition, within the subsample of rats undergone artificial stimulation, the stimulated limbs are always shifted toward negative values of PC1 approaching the convex hull of the control group. The same pattern is observed along PC2. At positive values of PC1, the diaphysis is characterized by a general increment in relative cortical thickness (up to 15%), with the exception of a region at the midshaft on the medial border, not corresponding to any muscle, which is characterized by a drastic reduction of relative cortical thickness (Figure 7 and Figure S2). The variation along PC2 is not related to the variable stimulation and it describes variation of relative cortical thickness mainly at the entire proximal region and on the lateral anterior margin of the distal portion of the diaphysis. At negative values of PC2, these regions are characterized by a reduction in relative cortical thickness; the opposite pattern is observed at positive values of PC2 (Figure 7 and Figure S2).

### 3.4 V.E.R.A. 1.0 and 2.0 Methods

**FIGURE 8:**
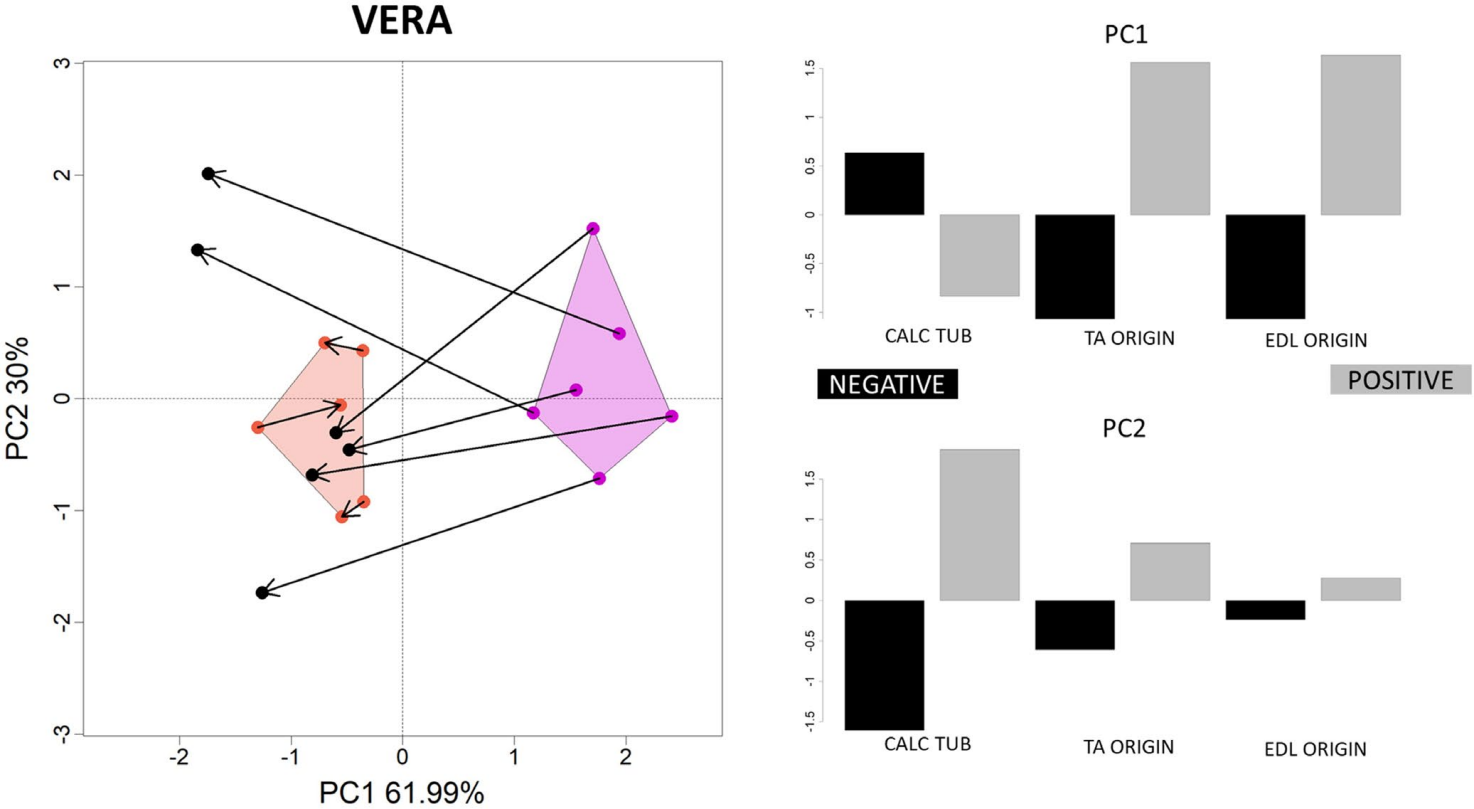
Principal Component Analysis of three unadjusted entheseal surface areas (in mm^2^). These include the calcaneal tuberosity (“CAL TUB”), which corresponds to muscles not stimulated during the experiment, and two entheses of muscles that were stimulated: the origin sites of tibialis anterior (“TA ORIGIN”) and extensor digitorum longus (“EDL ORIGIN”). The arrows represent links from the left to the right anatomical side. Stimulated rat hindlimbs are reported in magenta, control groups in dark orange. The PC loadings of each variable are presented on the right side of the figure. The two entheses of muscles involved in the experiment were proportionally larger in the stimulated limbs, which also showed substantial asymmetry.

When using V.E.R.A. 1.0's unadjusted entheseal measurements (Karakostis, Jeffery, and Harvati 2019), our PCA revealed a pronounced asymmetry in entheseal surface areas of stimulated hindlimbs compared to controls (Figure 8), with asymmetry consistently lower in the control specimens (shown in dark orange). The TA and EDL attachment sites in the stimulated limbs exhibit relatively larger surface areas, a pattern not observed in the control group. This finding highlights the impact of mechanical loading on 3D entheseal morphology (in line with previous research on the same sample and dataset; Karakostis, Jeffery, and Harvati 2019). The multivariate regression of the PC scores (until 90% of the variance) highlights a statistically significant relation with the variable “Stimulation” (*R* ^2^ = 0.60, *p*‐value = 0.001).

**FIGURE 9:**
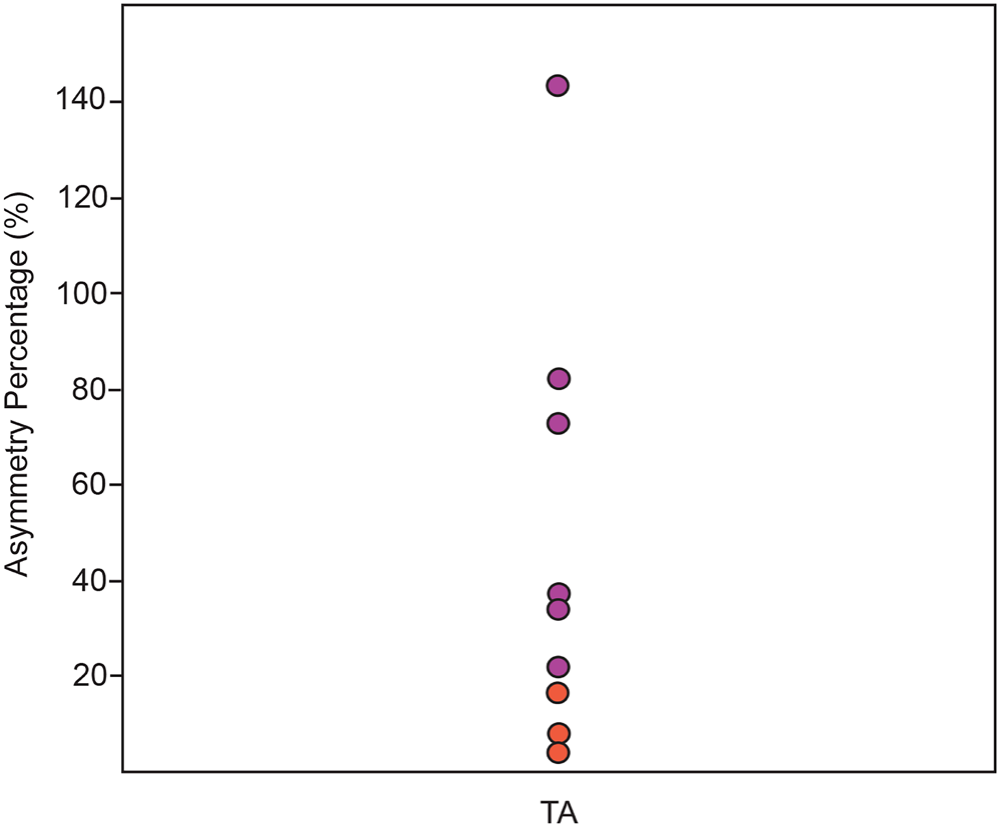
Percentage asymmetry in 3D entheseal changes within the tibialis anterior (TA) ridge, as identified using the semi‐automated V.E.R.A. 2.0 protocols (Karakostis 2025a; Karakostis 2025b; https://dx.doi.org/10.17504/protocols.io.5jyl82z8dl2w/v3). All six stimulated specimens (in magenta) consistently show higher asymmetry compared to the three controls (in dark orange). The data are visualized using a jitter plot.

Similarly, the simpler V.E.R.A. 2.0 analysis focusing on semi‐automatically identified 3D entheseal changes within the TA's ridge showed that the percentage of asymmetry was consistently higher in all stimulated specimens compared to controls (Figure 9).

## 4 Discussion

This study represents a laboratory‐based experimental demonstration that asymmetrical mechanical loading can create observable skeletal asymmetries within the same individual. By leveraging controlled conditions of asymmetrical muscle stimulation in strain and age‐matched Wistar rats, we effectively tested four methods: 3D geometric morphometrics, cross‐sectional geometry, morphometric mapping of cortical thickness, and the V.E.R.A. methods (1.0 and 2.0) to assess the ability of skeletal morphology to reflect habitual loading patterns, specifically lateralized loading.

Our findings reveal distinct asymmetrical skeletal responses under asymmetrical loading conditions. The 3D geometric morphometric analysis demonstrated that stimulated limbs showed periosteal expansion distally and contraction medio‐anteriorly at midshaft, while the cross‐sectional geometry analyses indicated enhanced biomechanical strength (represented by higher polar second moment of area) at the distal diaphysis in stimulated limbs. These results are in agreement with the predicted distribution of strain after applying finite element analysis on the same collection published by Vickerton and colleagues (Vickerton et al. 2014). Additionally, morphometric mapping of relative cortical thickness highlighted localized increases in the distal cortical thickness along the medial‐posterior diaphysis. The analysis of 3D entheseal surfaces using the V.E.R.A. 1.0 and 2.0 methods successfully distinguished between stimulated and unstimulated limbs within the same individuals, pointing to muscles that were actually stimulated during the experiment. Together, these results provide compelling evidence for the skeletal reflection of habitual asymmetric loading within an individual and highlight each approach's unique strengths in detecting and characterizing such asymmetry in dry skeletal remains.

### 4.1 What Is the Most Effective Method for Measuring and Describing Lateralization?

There is not a unique method for both evaluating and assessing lateralization from skeletal elements. In the case study presented here, CSG reveals significant differences in cross‐sectional strength between stimulated and non‐stimulated hindlimbs only at the distal portion of the diaphysis, in agreement with the results from relative cortical thickness. Interestingly, there are not significant differences in the midshaft, suggesting that traditional protocols in which only a single cross‐section is analyzed (usually at the midshaft) may fail in detecting lateralization patterns. For this reason, we suggest extending the analysis through the entire diaphysis. Nowadays, a number of open access methodological tools (e.g., morphomap and BoneJ) (Doube et al. 2010; Profico, Bondioli, et al. 2021) are available to easily automate the extraction of the biomechanical parameters along the diaphysis.

The analysis of the entire diaphysis by applying geometric morphometrics is able to capture general differences in shape variations but it is not capable of separating stimulated from non‐stimulated hindlimbs. On the contrary, if geometric morphometrics is applied in specific regions, the observed shape variations clearly intercept the morphological changes associated with asymmetry in mechanical loading. However, experimental laboratory animal databases generated under controlled conditions are far from the real application of reconstructing behaviors from archaeological and paleontological contexts. For this objective, we recommend that future anthropological studies begin with an initial analysis for detecting variations in relative cortical thickness between antimeric bones across the diaphysis and relying on this information to select the regions of interest (i.e., those showing patterns of lateralization). These regions may then be analyzed using CSG and geometric morphometrics at the same levels (intervals along biomechanical length), to measure and describe the pattern of asymmetry from both a biomechanical and morphological perspective, respectively. Lastly, the use of the V.E.R.A. methods (1.0 and 2.0) can provide a deeper understanding of the functional implications of the observed skeletal asymmetry by highlighting intra‐ and inter‐individual variation in muscle attachment areas and inferred patterns of asymmetrical muscle use.

It is worth noting that relying on separate methodologies for the study of muscle attachment areas and for the calculation of biomechanical parameters allows for a better understanding of bone adaptations in response to mechanical loading and helps overcome the technical issues in calculating cross‐sectional geometry parameters in diaphyseal portions corresponding to entheseal areas (due to local shape irregularity). In addition, our results showed that the more pronounced differences in relative cortical thickness and CSG between stimulated and unstimulated limbs did not strictly coincide with muscle attachment areas, suggesting that different loading factors may underlie the variation found in entheseal 3D surfaces (V.E.R.A.) and cross‐sectional morphology.

### 4.2 Limitations of the Study and Future Perspectives

The main limitation of this experimental study is its very small sample size, consisting of only nine pairs of hindlimbs from laboratory animals (including three control pairs). Nevertheless, to our knowledge, the use of a laboratory sample providing the opportunity to explore the effects of mechanical loading in animals with known asymmetry in applied forces is unprecedented in the fields of functional morphology and biological anthropology. The rare design of this experiment allowed us to control as much as possible for other factors of interindividual morphological variation, thereby isolating the effects of asymmetrical mechanical loading. Furthermore, in this experimental study, we analyzed in detail the effect of loading representing muscular action. Future research efforts should expand on these findings by applying the outlined methods to anthropological skeletal remains and additional animal‐based experiments. Such studies should also consider other factors of mechanical loading (e.g., gravitational/ground forces and/or various specific physical activity behaviors), quantifying the symmetric and asymmetric components of bone modeling and remodeling. For instance, future laboratory studies could specifically investigate the rate of bone modeling and skeletal adaptation by comparing juvenile and adult individuals exposed to the same experimental conditions, clarifying how exactly developmental age influences bone responsiveness.

Moreover, applying the combined methodology presented in this study to identified anthropological collections could significantly enhance the reliability of inferring habitual physical behaviors in bioarchaeological contexts (Blackburn and Knsel 2006; Steele and Mays 1995), yielding valuable insights into the daily lives and environmental interactions of past human and animal populations. Evidence from sports medicine (e.g., Haapasalo et al. 1996) indicates that repetitive loading during growth produces more pronounced skeletal modeling. On this basis, the future analysis of adequately documented anthropological collections (e.g., Karakostis and Hotz 2024; Sorrentino et al. 2025) from different contexts and time periods could help biological anthropologists assess how skeletal asymmetry relates to the magnitude and/or type of repetitive mechanical loading, the developmental timing of activity onset, and their complex interactions.

Overall, the pioneering and integrative approach proposed in this study, capable of analyzing both external and internal bone morphology, offers a robust basis for reconstructing key habitual asymmetrical behaviors in the past, such as for example the emergence and evolution of hand preference in the hominin lineage (e.g., Frayer et al. 2010, 2012; Lozano et al. 2017, 2009; Uomini 2009). In this context, the open access nature of the morphomap R package (Profico, Bondioli, et al. 2021) and semi‐automated V.E.R.A. 2.0 protocols (Karakostis 2025b) could greatly facilitate future applications of the proposed approach to bioarchaeological and paleoanthropological materials.

## Author Contributions

**Antonio Profico:** conceptualization, methodology, formal analysis, project administration, writing – original draft, writing – review and editing, investigation, software, data curation. **Nathan Jeffery:** conceptualization, methodology, resources, writing – review and editing, investigation, data curation. **Fotios Alexandros Karakostis:** conceptualization, methodology, formal analysis, project administration, writing – original draft, writing – review and editing, investigation, software, data curation.

## Funding

We acknowledge support from the Open Access Publishing Fund of the University of Tübingen.

## Ethics Statement

All in vivo animal work carried out in the original study was approved by the Home Office (PPL 40/3280) and conducted in strict accordance with the Animals (Scientific Procedures) Act of 1986.

## Conflicts of Interest

The authors declare no conflicts of interest.

## Supporting information


**Data S1:** ajpa70176‐sup‐0001‐Supinfo.docx.

## Data Availability

The data that support the findings of this study are openly available in Zenodo at https://zenodo.org/records/15283124, reference number https://10.5281/zenodo.15283124.
