# Supplementary material for: Can Asymmetrical Mechanical Loading Be Accurately Inferred From the Analysis of Skeletal Material?
Source: Am J Biol Anthropol. 2025 Dec 6;188(4):e70176. doi: 10.1002/ajpa.70176 (PMC12680966; doi:10.1002/ajpa.70176)
Supplement: Supplementary file 1 — Data S1: ajpa70176‐sup‐0001‐Supinfo.docx. [file AJPA-188-e70176-s001.docx]

**Supplementary information:**

- Supplementary figure 1. Geometric morphometrics: PC1 vs PC4.
- Supplementary figure 2. Relative cortical thickness variations.
- Supplementary table 1. Standardized cross-sectional parameters calculated (20%).
- Supplementary table 2. Standardized cross-sectional parameters calculated (50%).
- Supplementary table 3. Standardized cross-sectional parameters calculated (80%).

**
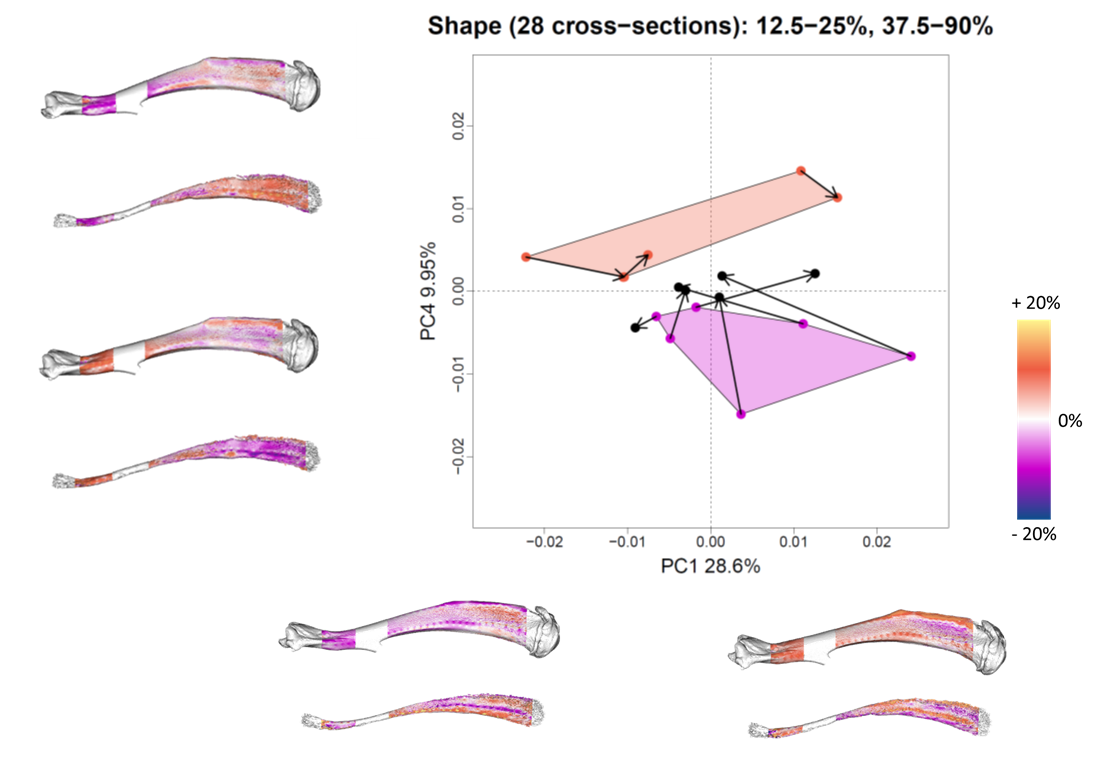
**

**Supplementary figure 1.** Principal component plot of the diaphyseal shape. Stimulated rat hindlimbs are reported in magenta, control groups in dark orange. The arrows represent links from left to the right side. Shape variations are reported at the extreme values of PC1 and PC4. Warm and cold colour palettes represent respectively the region characterized by local expansion and reduction of surface area.


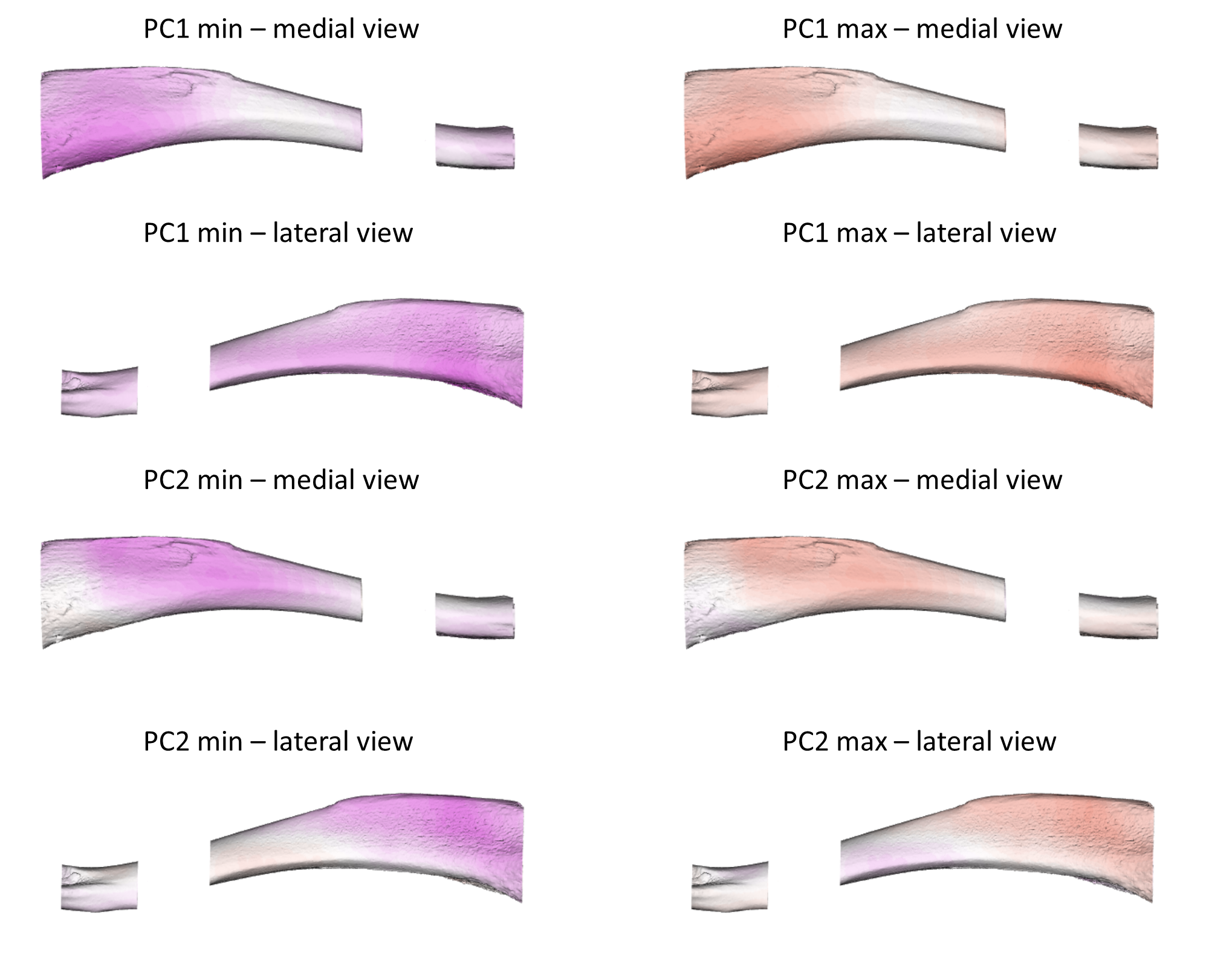


**Supplementary figure 2.** Relative cortical variations mapped onto a reference 3D model. Warm and cold colour palettes represent respectively the region characterized by a relative increment and decrement of cortical thickness.

**Supplementary table 1.** Standardized cross-sectional parameters calculated at 20% of the biomechanical length. Standardization: areas were divided by biomechanical length² and multiplied by 10^5^; perimeters were divided by biomechanical length and multiplied by 10^2^; moments of inertia were divided by biomechanical length⁴ and multiplied by 10⁹.

| T_area | M_area | CA | Ext_perim | Med_perim | Imin | Imax | J | stimulation |
| --- | --- | --- | --- | --- | --- | --- | --- | --- |
| 481.0319 | 76.26134 | 398.2375 | 25.22918 | 10.14694 | 1355.302 | 2113.697 | 3678.876 | N |
| 546.9029 | 66.46067 | 478.8645 | 27.99558 | 9.582246 | 1666.41 | 2983.882 | 5053.755 | Y |
| 430.2979 | 70.68365 | 368.6747 | 22.92273 | 9.532819 | 1184.793 | 1932.687 | 3064.816 | N |
| 519.7812 | 82.00162 | 430.741 | 26.20498 | 10.90101 | 1503.986 | 2658.814 | 4425.999 | N |
| 409.1463 | 49.78366 | 358.1783 | 23.55895 | 8.672824 | 1090.94 | 1416.4 | 2707.978 | N |
| 381.9691 | 58.60914 | 331.3028 | 22.25484 | 8.825877 | 911.6935 | 1565.216 | 2429.764 | N |
| 485.1999 | 78.39601 | 400.138 | 24.59782 | 9.930893 | 1437.75 | 2076.289 | 3723.286 | N |
| 481.6566 | 51.67891 | 429.0264 | 25.40752 | 8.650527 | 1321.83 | 2245.256 | 3870.435 | N |
| 437.6983 | 59.48019 | 387.134 | 24.64173 | 8.633081 | 1155.825 | 2133.86 | 3226.319 | N |
| 447.9843 | 59.25098 | 383.2571 | 24.34948 | 8.839853 | 1315.035 | 1710.69 | 3195.766 | N |
| 455.0851 | 38.24208 | 416.6211 | 25.24429 | 7.37873 | 1283.269 | 1867.985 | 3411.815 | Y |
| 547.3575 | 84.81528 | 473.9443 | 26.10687 | 10.44615 | 1762.726 | 3414.508 | 5074.794 | Y |
| 381.8612 | 35.15497 | 342.8313 | 21.91892 | 7.112687 | 960.4458 | 1245.415 | 2321.593 | N |
| 505.1619 | 61.719 | 441.9645 | 26.58521 | 9.535606 | 1682.666 | 2149.827 | 4136.536 | Y |
| 521.7178 | 75.30466 | 457.1472 | 27.30297 | 11.15883 | 1561.52 | 3243.814 | 4707.68 | Y |
| 481.6181 | 45.23178 | 431.4531 | 24.50308 | 7.569164 | 1404.008 | 2122.219 | 3736.07 | Y |
| 456.0012 | 43.15711 | 412.3124 | 24.89609 | 8.357102 | 1117.881 | 2133.729 | 3545.003 | N |
| 425.1122 | 45.15542 | 388.3113 | 24.1653 | 7.840608 | 1148.369 | 1934.514 | 3040.587 | N |

**Supplementary table 2.** Standardized cross-sectional parameters calculated at 50% of the biomechanical length. Standardization: areas were divided by biomechanical length² and multiplied by 10^5^; perimeters were divided by biomechanical length and multiplied by 10^2^; moments of inertia were divided by biomechanical length⁴ and multiplied by 10⁹.

| T_area | M_area | CA | Ext_perim | Med_perim | Imin | Imax | J | stimulation |
| --- | --- | --- | --- | --- | --- | --- | --- | --- |
| 496.8269 | 118.4696 | 365.6903 | 25.8474 | 12.72752 | 1472.38 | 2491.861 | 3601.542 | N |
| 537.1223 | 88.40219 | 400.3248 | 26.14532 | 11.3791 | 1994.443 | 2337.78 | 3708.802 | Y |
| 464.0483 | 91.30887 | 395.5229 | 25.08877 | 10.66399 | 1220.287 | 2373.073 | 3934.642 | N |
| 502.695 | 103.5223 | 385.4693 | 25.57574 | 11.68107 | 1594.449 | 2506.82 | 3713.107 | N |
| 477.4399 | 90.31676 | 344.7569 | 25.65627 | 11.15889 | 1325.759 | 2150.884 | 2993.719 | N |
| 421.5328 | 81.24357 | 361.0573 | 24.67893 | 10.31198 | 1047.398 | 1899.393 | 3227.449 | N |
| 491.0719 | 103.3658 | 374.4409 | 25.76227 | 11.49354 | 1477.263 | 2436.559 | 3543.908 | N |
| 574.4881 | 90.04712 | 432.429 | 26.46409 | 11.3679 | 1951.295 | 3101.002 | 4349.413 | N |
| 466.8308 | 84.90627 | 405.1385 | 25.38717 | 10.96923 | 1385.24 | 2195.206 | 3927.297 | N |
| 447.9435 | 85.27412 | 350.0802 | 24.7059 | 10.66577 | 1405.308 | 1857.749 | 2958.844 | N |
| 486.3893 | 71.13444 | 370.9356 | 24.88314 | 10.14629 | 1519.852 | 2075.434 | 3085.642 | Y |
| 521.5395 | 94.56662 | 452.9197 | 26.04463 | 10.98462 | 1900.002 | 2416.637 | 4739.704 | Y |
| 441.757 | 76.5541 | 352.3785 | 24.20074 | 10.11842 | 1232.663 | 2009.457 | 2935.16 | N |
| 506.1711 | 83.94511 | 376.6548 | 26.19534 | 11.08534 | 1765.023 | 2072.238 | 3292.868 | Y |
| 454.1389 | 96.05318 | 380.0996 | 25.60013 | 11.41703 | 1149.007 | 2251.561 | 3724.584 | Y |
| 517.1033 | 98.93013 | 403.667 | 26.41337 | 11.38673 | 1754.457 | 2564.499 | 3914.903 | Y |
| 559.177 | 94.82057 | 414.1288 | 25.95155 | 11.29316 | 1980.555 | 2717.286 | 4032.362 | N |
| 462.668 | 81.4114 | 404.3794 | 25.10462 | 10.64879 | 1352.533 | 2142.727 | 3844.47 | N |

**Supplementary table 3.** Standardized cross-sectional parameters calculated at 80% of the biomechanical length. Standardization: areas were divided by biomechanical length² and multiplied by 10^5^; perimeters were divided by biomechanical length and multiplied by 10^2^; moments of inertia were divided by biomechanical length⁴ and multiplied by 10⁹.

| T_area | M_area | CA | Ext_perim | Med_perim | Imin | Imax | J | stimulation |
| --- | --- | --- | --- | --- | --- | --- | --- | --- |
| 1179.03 | 681.6115 | 418.2516 | 42.51564 | 30.43657 | 6417.129 | 8168.547 | 12484.07 | N |
| 898.5268 | 424.1729 | 508.0072 | 37.05391 | 24.20231 | 3816.821 | 8049.121 | 12997.78 | Y |
| 977.0733 | 499.8282 | 466.8096 | 38.25384 | 26.75277 | 4410.543 | 8461.54 | 11642.48 | N |
| 1135.493 | 548.9473 | 504.3352 | 40.83921 | 28.29908 | 6487.787 | 9099.78 | 13383.22 | N |
| 929.6348 | 494.4809 | 467.6199 | 37.5728 | 26.56162 | 3953.971 | 7499.203 | 12554.65 | N |
| 879.3691 | 500.8805 | 371.8652 | 37.10063 | 25.13528 | 3599.556 | 6345.014 | 9011.601 | N |
| 1168.036 | 667.6202 | 421.563 | 41.42128 | 31.19041 | 6487.922 | 7991.406 | 12394.62 | N |
| 1086.268 | 567.9306 | 556.6908 | 40.86147 | 28.78133 | 6016.324 | 8881.825 | 16388.94 | N |
| 993.273 | 510.9726 | 471.8471 | 39.79669 | 25.7266 | 4853.948 | 8693.205 | 12245.83 | N |
| 1064.71 | 538.8765 | 450.088 | 39.77999 | 27.84255 | 5545.958 | 7605.432 | 11271.76 | N |
| 939.1233 | 455.7148 | 518.0589 | 36.11873 | 24.94868 | 4809.22 | 7095.803 | 13081.26 | Y |
| 1097.2 | 560.8591 | 524.5999 | 39.065 | 27.60504 | 6153.187 | 9326.099 | 14017.88 | Y |
| 994.9451 | 471.3796 | 450.9966 | 38.99586 | 26.76161 | 5131.308 | 7284.735 | 10666.91 | N |
| 966.4509 | 479.1834 | 522.4944 | 38.45897 | 25.97509 | 4674.88 | 8120.443 | 14037.33 | Y |
| 859.1832 | 496.8583 | 356.259 | 36.62003 | 25.20059 | 3099.199 | 6276.988 | 8502.691 | Y |
| 1001.781 | 502.1806 | 428.0627 | 38.24261 | 26.96176 | 4809.172 | 7236.226 | 10353.69 | Y |
| 1052.229 | 597.0072 | 490.3908 | 40.70067 | 29.22073 | 5306.92 | 8124.212 | 14730.5 | N |
| 1004.651 | 536.1687 | 458.9589 | 39.48026 | 26.59891 | 4893.679 | 8137.337 | 11804.26 | N |
